# Risk factors for relaparotomy after a cesarean delivery: a case-control study

**DOI:** 10.1186/s12884-024-06455-6

**Published:** 2024-04-17

**Authors:** Uri Amikam, Yael Botkovsky, Alyssa Hochberg, Aviad Cohen, Ishai Levin, Yariv Yogev, Liran Hiersch, Anat Lavie

**Affiliations:** 1 Department of Obstetrics and Gynecology, Lis Hospital for Women’s Health, Tel Aviv Sourasky Medical Center, 6 Weizmann St, Tel Aviv, Israel; 2 Faculty of Medicine, Tel Aviv University, Tel Aviv, Israel; 3 Helen Schneider Hospital for Women, Rabin Medical Center, Petach Tikva, Israel

**Keywords:** Cesarean delivery, Maternal complications, Relaparotomy, Maternal morbidity, Placental abruption, Hypertensive disorders of pregnancy

## Abstract

**Background:**

Relaparotomy following a cesarean delivery (CD) is an infrequent complication, with inconsistency regarding risk factors and indications for its occurrence. We therefore aimed to determine risk factors and indications for a relaparotomy following a CD at a single large tertiary center.

**Methods:**

A retrospective case-control single-center study (2013–2023). We identified all women who had a relaparotomy up to six weeks following a CD (study group). Maternal characteristics, obstetrical and surgical data were compared to a control group in a 1:2 ratio. Controls were women with a CD before and immediately after each case in the study group, who did not undergo a relaparotomy. Included were CDs occurring after 24 gestational weeks. CD performed at different centers and indications for repeat surgery unrelated to the primary surgery (e.g., appendicitis) were excluded. Logistic regression was used to adjust for potential confounders.

**Results:**

During the study period, 131,268 women delivered at our institution. Of them, 28,280 (21.5%) had a CD, and 130 patients (0.46%) underwent a relaparotomy. Relaparotomies following a CD occurred during the first 24 h, the first week, and beyond the first week, in 59.2%, 33.1%, and 7.7% of cases, respectively. In the multivariable logistic regression analysis, relaparotomy was significantly associated with Mullerian anomalies (aOR 3.33, 95%CI 1.08–10.24, *p* = 0.036); uterine fibroids (aOR 3.17, 95%CI 1.11–9.05,*p* = 0.031); multiple pregnancy (aOR 4.1, 95%CI 1.43–11.79,*p* = 0.009); hypertensive disorders of pregnancy (aOR 3.46, 95%CI 1.29–9.3,*p* = 0.014); CD during the second stage of labor (aOR 2.54, 95%CI 1.15–5.88, *p* = 0.029); complications during CD (aOR 1.62, 95%CI 1.09–3.21,*p* = 0.045); and excessive bleeding during CD or implementation of bleeding control measures (use of tranexamic acid, a hemostatic agent, or a surgical drain) (aOR 2.23, 95%CI 1.29–4.12,*p* = 0.012). Indications for relaparotomy differed depending on the time elapsed from the CD, with suspected intra-abdominal bleeding (36.1%) emerging as the primary indication within the initial 24 h.

**Conclusion:**

We detected several pregnancy, intrapartum, and intra-operative risk factors for the need for relaparotomy following a CD. Practitioners may utilize these findings to proactively identify women at risk, thereby potentially reducing their associated morbidity.

**Supplementary Information:**

The online version contains supplementary material available at 10.1186/s12884-024-06455-6.

## Background

Cesarean delivery (CD) is the most common obstetrical surgery, with a rising incidence worldwide, increasing from approximately 12% in the year 2000 to 22–25% in 2018 [1, 2] and reaching more than 32% in the United States in 2021 [3], making it one of the most common operations performed worldwide.

Although CD is considered a safe procedure, it confers a two-fold higher risk for severe maternal morbidity compared to vaginal delivery [4]. Amongst the maternal short-term complications after CD is hemorrhage, need for blood products transfusion, intra-abdominal infection, and injury to adjacent organs [5–7], which may warrant a repeat laparotomy (relaparotomy). Relaparotomy is defined as an abdominal operation performed after an initial surgery including skin opening [8, 9] and entrance into the abdominal cavity [10]. A relaparotomy has major implications for the patient and her family and necessitates separating the parturient from her newborn. It also confers potential high maternal morbidity and mortality [11, 12].

Data regarding relaparotomy after a CD is conflicting. In previous studies, the incidence of this complication ranges widely, with up to a more than ten-fold difference among various reports (0.07-0.9%) [5, 7, 12–18]. Moreover, data demonstrates conflicting results regarding risk factors for the need for repeat surgery. For example, while some described multiple pregnancies as a risk factor [7, 16], others did not find such an association [5, 14]. Furthermore, some studies were limited only to re-surgeries due to excessive bleeding [9, 19]. In addition, previous studies defined the time from the initial CD to the relaparotomy differently, with some limited to the same hospitalization as the original CD [13, 20], whilst others included cases occurring up to one week [7, 9], and even six weeks [5], after the CD. Additionally, there is a paucity of data regarding the indications for relaparotomy following CD stratified by the time interval from the CD to the repeated surgery, with only a few studies [5, 15] elaborating on it. Lastly, most of the previous studies comprised a relatively small number of cases ranging between 18 and 64 [5, 7, 12–14, 16–18], with only one comprising 80 patients [15].

Due to the infrequency of this complication and the wide variability in reported data, our objective was to ascertain the incidence of a relaparotomy after a CD and to identify risk factors for its occurrence, at a single tertiary center. Our secondary aim was to describe the indications for the relaparotomy according to the time elapsed from the initial surgery.

## Methods

### Study population

We conducted a retrospective case-control study between January 2013 and October 2023 in a large single-tertiary, university-affiliated, medical center, with over 12,000 deliveries annually. The local institutional review board (IRB) approved the study (TLV-0618-22).

The study group comprised patients who had a CD at our institution and needed a relaparotomy for indications related to the CD within six weeks following delivery. Relaparotomy was defined as the need for a repeated surgical intervention which included opening of the skin and entrance into the abdominal cavity. For each patient in the study group, controls were compared in a 1:2 ratio. Controls were women with a CD at > 24 weeks of gestation before and after each case in the relaparotomy group who did not require a relaparotomy. By selecting women who had a CD before and after each case, we aimed to minimize confounders related to the specific conditions in the operating theatre. At our institution, all CDs are performed by two surgeons; at least one of whom is a senior obstetrician or a resident in the second half of his residency period. All relaparotomies are performed by senior obstetrician-gynecologist surgeons.

Included in the study group were women who underwent both surgeries (i.e., CD and a relaparotomy) at our institution, were > 24 weeks of gestation at the CD, and who underwent intra-peritoneal exploration at the relaparotomy. Exclusion criteria included cases where repeat surgery was performed due to an indication unrelated to the primary surgery (appendicitis, etc.), or if the repeated surgery was performed more than 6 weeks following the CD. The study group was further divided into three groups based on the time interval from the CD to the relaparotomy: early relaparotomy (within 24 h), intermediate relaparotomy (between one to seven days), and late relaparotomy (between 7 days and 6 weeks).

### Data collection

Medical records of all women who delivered at our institution during the defined study period were reviewed, and the patients who had a relaparotomy were identified. Patients’ data were anonymized and de-identified before the analysis. Data were extracted from the departmental electronic patient database and the operating records of the CD and relaparotomy surgeries. Antenatal follow-up test results and pregnancy outcomes are consistently recorded in the database during prenatal check-ups, upon admission for delivery, and postpartum.

Demographic and obstetric variables of the study cohort were recorded, including maternal age; pre-pregnancy body mass index (kg/m^2^); maternal comorbidities, including chronic hypertension, and thrombophilia; uterine fibroids; Mullerian anomalies, including bicornuate uterus, unicornuate uterus, didelphic uterus, and septate uterus; pregnancy achieved by assisted reproductive technology (ART); gravidity; parity; multiple pregnancy; aspirin treatment during pregnancy; low molecular weight heparin (LMWH) use during pregnancy; prior CD; and pregnancy complications, including hypertensive disorders of pregnancy (HDP), gestational diabetes mellitus (GDM), placenta previa, and placenta accreta spectrum (PAS). Delivery and surgery characteristics that were examined included: gestational age at delivery; preterm delivery (< 37 gestational weeks); neonatal birthweight; fever during labor (a temperature of 38.0 °C (100.4 °F) or higher taken at least twice at least 8 h apart); placental abruption (suspected on a clinical basis and confirmed on placental pathology examination); amniotic fluid color; time of day of the CD; surgeon’s experience and intra-operative findings, including surgery duration, CD defined as complicated (including intraabdominal adhesions, uterine incision extensions, calling for assistance during surgery, or bladder injury), or CD with excessive bleeding or use of bleeding control measures (including estimated blood loss (EBL) ≥ 1000 ml, tranexamic acid (TXA) use during surgery, use of a hemostatic agent (such as Gelfoam®, and Surgicell®), and use of surgical suction drain. EBL was assessed clinically during the CD and recorded in the operation room (OR) notes. Any intra-abdominal adhesions observed were recorded and described in the OR report. In the study group, the indication for relaparotomy, the time interval from the CD, and the relaparotomy findings were recorded.

The indications for CD were divided into elective or urgent. An elective CD was defined as a scheduled CD performed for maternal, fetal, or placental indications that posed a risk for vaginal delivery, such as a placenta previa or non-vertex presentation. An urgent CD was defined as a non-elective CD, including all CDs that occurred during labor and delivery, such as those due to labor dystocia, or a non-reassuring fetal heart.

Indications for the relaparotomy were divided into several categories, including suspected intra-abdominal bleeding; bleeding from the subcutaneous tissue; uterine atony and post-partum hemorrhage (PPH); infection, defined as sepsis, peritonitis, scar infection, or abscess formation; evisceration, defined as a defect in the integrity of the fascia; and suspected injury to adjacent organs, including the urinary tract system and the gastrointestinal system.

Post-surgery complications, which occurred after the CD or after the relaparotomy, were evaluated and included: maternal death; a thrombotic event in the post-partum period (up to 6 weeks following the CD); intensive care unit (ICU) admission; paralytic ileus; post-partum fever; need for readmission; and hospitalization length. The rate of future deliveries at our institution was also recorded until the end of the study period in October 2023.

### Statistical analysis

Parameters were compared between the study and control groups. Univariate analysis was performed using the Mann–Whitney *U* test, chi-square, and Fisher’s exact tests as appropriate. Multivariable logistic regression analysis was used to identify factors associated with relaparotomy after CD. Variables that were found to be significantly different between the groups (*p* < 0.05) in the univariate analysis entered the initial regression model. Differences were considered significant when the two-sided *P*-value was < 0.05. Statistical analysis was performed using SPSS 25.0 (IBM Corporation, Chicago, USA) software.

## Results

During the study period, 131,268 women delivered at our institution. Of them, 28,280 (21.5%) had a CD. Overall, 130 patients (0.46%) underwent relaparotomy after CD. These women were compared to a control group, which included 260 women, who had a CD before and after each case in the study group.

Maternal demographics and gestational characteristics are presented in Table 1. In the univariate analysis, relaparotomy after a CD, compared to the control group, was associated with the following: Mullerian anomalies (6.2% vs. 3.1%, *p* = 0.014); uterine fibroids (6.9% vs. 3.5%, *p* = 0.009); ART (27.7% vs. 10.4%, *p* < 0.001); multiple pregnancy (11.5% vs. 3.8%, *p* = 0.003); HDP (13.8% vs. 4.2%, *p* < 0.001); and LMWH use during pregnancy (9.2% vs. 3.8%, *p* = 0.03).

**Table 1:** Demographics and current gestation characteristics of the cohort

Variable	Relaparotomy group *N* = 130	Control group *N* = 260	*P*-value
Maternal age (years)	35.7 ± 4.7	34.2 ± 4.8	0.843
Pre-pregnancy BMI (kg/m^2^)	23.9 ± 4.7	24.4 ± 4.9	0.354
GWG (kg)	12 ± 6	12 ± 5.5	0.386
Chronic hypertension	3 (2.3)	3 (1.2)	0.383
Inherited or acquired Thrombophilia	6 (4.6)	5 (1.9)	0.13
Pre-gestational Diabetes mellitus	3 (2.3)	5 (1.9)	0.801
Previous CD (≥ 1)	63 (48.5)	107 (41.2)	0.17
Mullerian anomalies*	8 (6.2)	8 (3.1)	**0.014**
Uterine fibroids	9 (6.9)	9 (3.5)	**0.009**
Gravidity	2.9 ± 1.7	2.4 ± 1.6	0.609
Parity	1.2 ± 1.2	1 ± 1.2	0.415
Primiparous	47 (36.2)	108 (40)	0.306
ART conception	36 (27.7)	28 (10.4)	**< 0.001**
Multiple pregnancy	15 (11.5)	10 (3.8)	**0.003**
GDM	27 (20.8)	37 (14.2)	0.107
HDP	18 (13.8)	11 (4.2)	**< 0.001**
Placenta previa	8 (6.2)	7 (2.7)	0.094
PAS	2 (1.5)	0	N/A
Aspirin use during pregnancy	15 (11.5)	22 (8.5)	0.34
LMWH use during pregnancy	12 (9.2)	10 (3.8)	**0.03**

Categorical variables are presented as numbers (percentages), and continuous variables are presented as mean ± standard deviation

Abbreviations: BMI – Body mass index; GWG – gestational weight gain; CD – Cesarean delivery; ART – Assisted reproductive technology; GDM – Gestational diabetes mellitus; HDP – Hypertensive disorders in pregnancy; PAS – Placenta accreta spectrum; N/A – Not applicable; LMWH – Low molecular weight heparin

* Mullerian anomalies – including any of the following: bicornuate uterus, unicornuate uterus, didelphic uterus, and septate uterus

The labor and CD characteristics are presented in Table 2. In the univariate analysis, relaparotomy after a CD, compared to the control group, was associated with the following: preterm labor (25.4% vs. 14.2%, *p* = 0.007); placental abruption (9.2% vs. 1.9%, *p* < 0.001); lower birthweight (2875 ± 799 gram vs. 3062 ± 642 gram, *p* = 0.015); CD during the second stage of labor (14.6% vs. 5.8%, *p* = 0.004); longer CD duration (41.5 ± 28.2 min vs. 30.4 ± 11.6 min, *p* < 0.001); increased rate of complicated CD (47.7% vs. 26.9%, *p* < 0.001); and an increased rate of CD with excessive bleeding or use of bleeding control measures (46.9% vs. 18.5%, *p* < 0.001). Regarding postoperative complications, which occurred after the CD or the relaparotomy, women in the study group had a higher incidence of ICU admission (43.8% vs. 0.8%, *p* < 0.001); paralytic ileus (6.2% vs. 0.4%, *p* < 0.001); post-partum fever (16.9% vs. 0.8%, *p* < 0.001); a longer duration of hospitalization (9.9 ± 7.9 days, 5.4 ± 4.1days, *p* < 0.001); and lower rates of future deliveries (6.2% vs. 22.3%, *p* < 0.001).

**Table 2:** Delivery, surgery, and hospitalization characteristics of the cohort

Variable	Relaparotomy group *N* = 130	Control group *N* = 260	*P*-value	
Gestational age (weeks)	37^5/7^ (3^0/7^)	38^2/7^(2^0/7^)	0.19	
Pre-term delivery	33 (25.4)	37 (14.2)	**0.007**	
Fever during labor	9 (6.9)	10 (3.8)	0.183	
Placental abruption	12 (9.2)	5 (1.9)	**< 0.001**	
Amniotic fluid			**0.032**	
Clear	99 (76.2)	221 (85)		
Other (meconium or bloody)	31 (23.8)	39 (15)		
Maternal hemoglobin prior to CD (g/dl)	12.2 ± 1.3	12.1 ± 1.1	0.224	
Maternal platelet count prior to CD	186.1 ± 62	202.8 ± 58.6	0.249	
Time of CD			0.286	
Morning (7–15)	62 (47.7)	112 (43.1)		
Evening (15–23)	45 (34.6)	111 (42.7)		
Nighttime (23 − 7)	23 (17.7)	37 (14.2)		
Birthweight (grams)^*^	2875 ± 799	3062 ± 642	**0.015**	
CD type			**0.044**	
Elective	60 (46.2)	148 (56.9)		
Urgent	70 (53.8)	122 (43.1)		
Primary surgeon			0.128	
Attending	48 (36.9)	117 (45)		
Resident	48 (36.9)	117 (45)		
Second stage CD	19 (14.6)	15 (5.8)	**0.004**	
General anesthesia	12 (9.2)	11 (4.2)	**0.048**	
Cesarean duration, minutes	41.5 ± 28.2	30.4 ± 11.6	**< 0.001**	
Calling for assistance	25 (19.2)	14 (5.4)	**< 0.001**	
Intra-abdominal adhesions	40 (30.8)	51 (19.6)	**0.014**	
Uterine incision extensions	27 (20.8)	18 (6.9)	**< 0.001**	
Bladder injury during the CD	0	2 (0.77)	N/A	
Complicated CD^**^	62 (47.7)	70 (26.9)	**< 0.001**	
EBL ≥ 1000 ml	23 (17.7)	14 (5.4)	**< 0.001**	
TXA use during the CD	18 (13.8)	7 (2.7)	**< 0.001**	
Use of a hemostatic agent	52 (40)	36 (13.8)	**< 0.001**	
Use of a surgical suction drain	11 (8.5)	2 (0.8)	**< 0.001**	
Excessive bleeding or the use of bleeding control measures ^***^	61 (46.9)	48 (18.5)	**< 0.001**	
Tubectomy/tubal ligation	7 (5.4)	10 (3.8)	0.413	
Suture of the uterus intra-abdominally	6 (4.6)	10 (3.8)	0.649	
Packed red blood cell transfusion	91 (70)	4 (1.5)	**< 0.001**	
Cesarean hysterectomy	1 (0.8)	0	N/A	
Uterine rupture	2 (1.5)	0	N/A	
Hysterectomy (excluding cesarean hysterectomy)	13 (10)	0	**< 0.001**	
Maternal death	0	0	N/A	
DIC	38 (29.2)	0	**< 0.001**	
ICU admission	57 (43.8)	2 (0.8)	**< 0.001**	
Thrombotic event	3 (2.31)	1 (0.4)	0.126	
Paralytic ileus	8 (6.2)	1 (0.4)	**< 0.001**	
Postpartum fever	22 (16.9)	2 (0.8)	**< 0.001**	
Duration of hospitalization, days	9.9 ± 7.9	5.4 ± 4.1	**< 0.001**	
Readmission	33 (25.4)	7 (2.7)	**< 0.001**	
Future delivery at our institution	8 (6.2)	58 (22.3)	**< 0.001**	

Categorical variables are presented as numbers (percentages), and continuous variables are presented as mean (± standard deviation)

Abbreviations: CD – Cesarean delivery; LGA – Large for gestational age; SGA – Small for gestational age; EBL – Estimated blood loss; TXA – Tranexamic acid; N/A – Not applicable; DIC – Disseminated intravascular coagulation; ICU – Intensive care unit

* Study group – 145 neonates, control group – 270 neonates

** Including any of the following– intra-abdominal adhesions, uterine incision extensions, calling for assistance, and bladder injury

*** Including any of the following– EBL ≥ 1000, TXA use during CD, use of a hemostatic agent, and use of a surgical suction drain

Multivariable logistic regression examining risk factors associated with a relaparotomy following a CD is presented in Supplementary Table 1. The risk factors found were Mullerian anomalies (adjusted odds ratio (aOR) 3.33, 95% CI 1.08–10.24, *p* = 0.036); uterine fibroids (aOR 3.17, 95% CI 1.11–9.05, *p* = 0.031); pregnancy conceived by ART (aOR 4.8, 95% CI 2.28–10.1, *p* < 0.001); multiple pregnancy (aOR 4.1, 95% CI 1.43–11.79, *p* = 0.009); HDP (aOR 3.46, 95% CI 1.29–9.3); placental abruption (aOR 4.62, 95% CI 1.09–19.59, *p* = 0.038); CD during the second stage of labor (aOR 2.54, 95% CI 1.1–5.88, *p* = 0.029); CD duration (aOR 1.12, 95% CI 1.1–1.3, *p* = 0.048); complicated CD (aOR 1.62, 95% CI 1.09–3.21, *p* = 0.045); and excessive bleeding or use of bleeding control measures (aOR 2.23, 95% CI 1.29–4.12, *p* = 0.012). Notably, LMWH use during pregnancy, pre-term delivery, non-clear amniotic fluid, and neonatal birthweight, were not found to be statistically significant in the logistic regression model.

The median time from the CD to the relaparotomy was 17.5 h (IQR 5-58.3), and 59.2% of the relaparotomy cases occurred in the first 24 h after the CD, with only 7.7% occurring between one week and up to 6 weeks after the initial surgery Additionally, the mean duration of relaparotomy was 68.8 (± 49.6 standard deviation) minutes, and nine patients (6.9%) needed a second relaparotomy (data not shown in the Tables).

Table 3 presents the indications for the relaparotomy, and stratification by the time interval from the CD. The main indication for relaparotomy was suspected intra-abdominal bleeding (45.4%). Nine patients (6.9%) needed a second relaparotomy, and 13 patients (10%) underwent a hysterectomy in the first or second relaparotomy. There were no women who underwent a third relaparotomy. In the early period, the main indication was suspected intra-abdominal bleeding (36.1%); in the intermediate period, it was scar disruption (10%); and in the late period, the main indications were infection and GI injury/bowel obstruction (3.1% each).

**Table 3:** Indications for the relaparotomy following the cesarean delivery and stratification by the time interval to relaparotomy

Parameter	Study group (*N* = 130)
Indication for relaparotomy	
Abdominal bleeding	59 (45.4)
Uterine atony	20 (15.4)
Scar disruption	13 (10)
Subcutaneous hematoma	10 (7.7)
GI injury/bowel obstruction	10 (7.7)
Infection	9 (6.9)
Genitourinary injury	8 (6.2)
Foreign body	1 (0.8)
Early (< 24 hours)	77 (59.2)
Abdominal bleeding	47 (36.1)
Uterine atony	19 (14.6)
Subcutaneous hematoma	7 (5.4)
Genitourinary injury	4 (3.1)
Intermediate (1 day-7 days)	43 (33.1)
Scar disruption	13 (10)
Abdominal bleeding	12 (9.2)
GI injury/bowel obstruction	6 (4.6)
Infection	5 (3.8)
Subcutaneous hematoma	3 (2.3)
Genitourinary injury	3 (2.3)
Foreign body	1 (0.8)
Late (7 days-42 days)	10 (7.7)
Infection	4 (3.1)
GI injury/bowel obstruction	4 (3.1)
Genitourinary injury	1 (0.8)
Uterine atony	1 (0.8)

Categorical variables are presented as numbers (percentages)

Abbreviations: GI – Gastrointestinal

**Fig. 1:**
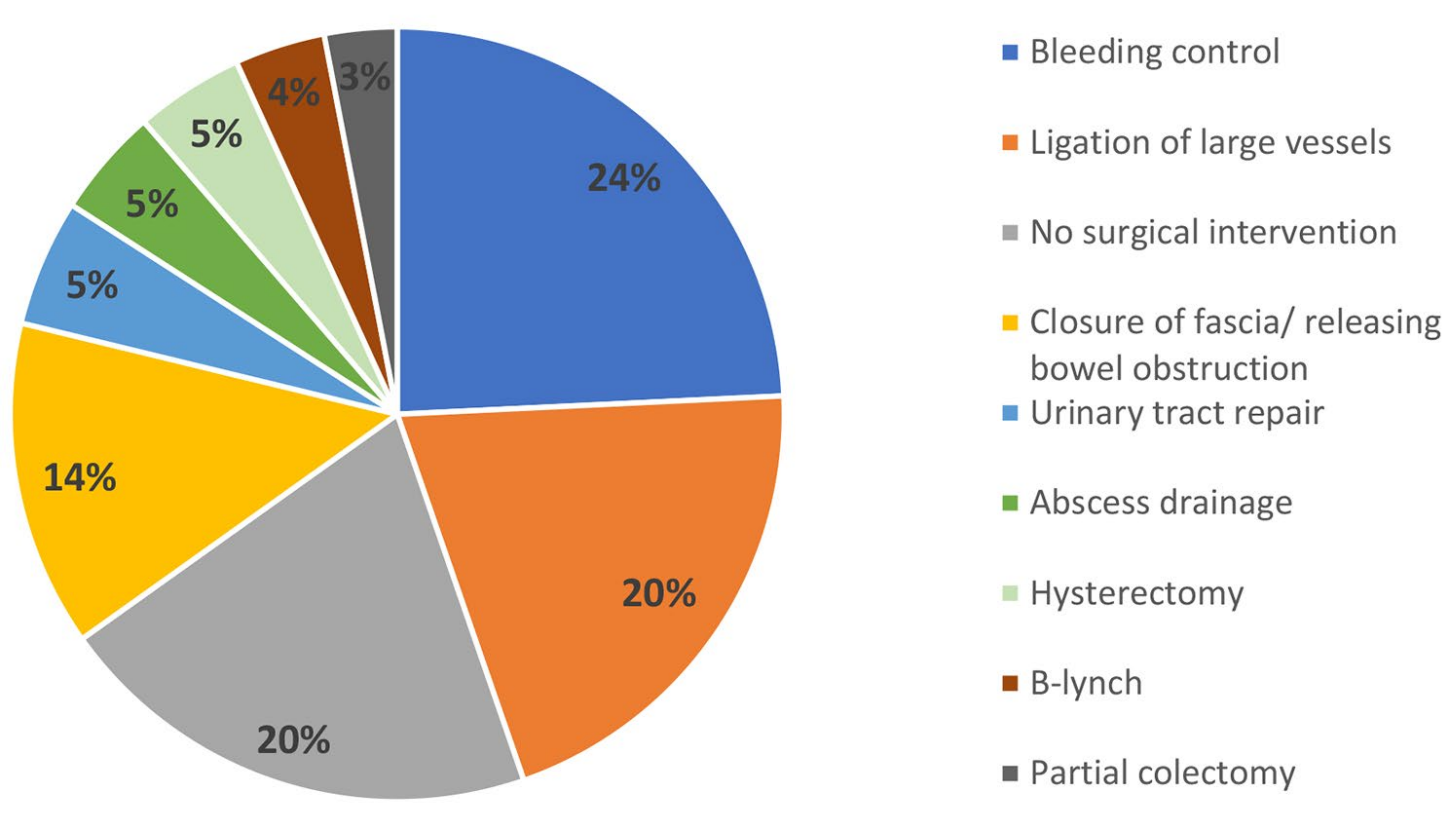
Surgical interventions performed during the relaparotomy * *N* = 130 cases, in two cases there was more than one intervention ** In seven cases, a hysterectomy was performed during the second relaparotomy

Figure 1 presents the relaparotomy intervention that was performed. As depicted, the most common intervention was bleeding control including electrocautery and surgical sutures, and the second most common intervention was ligation of the large vessels including the uterine and iliac arteries.

**Fig. 2:**
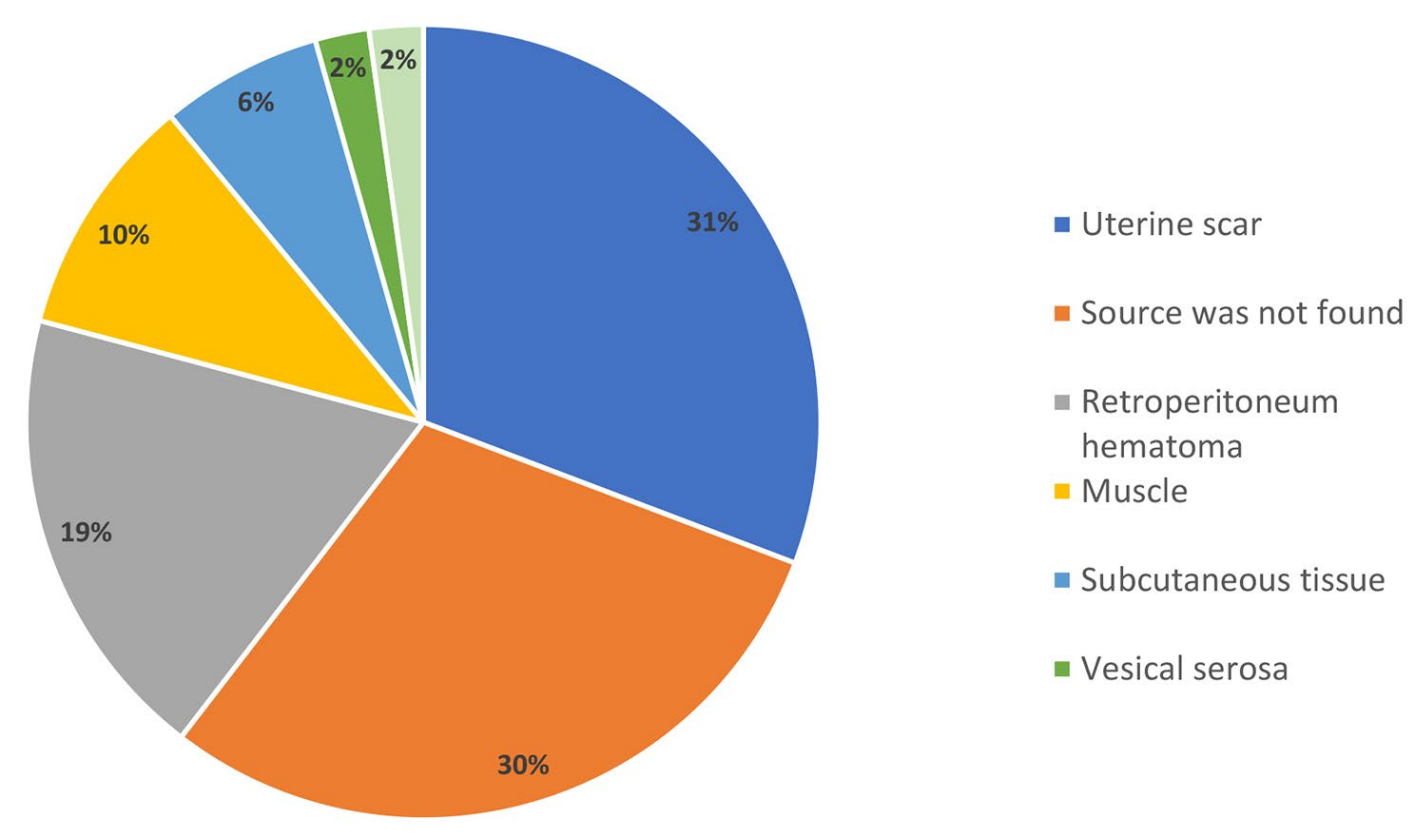
The source of bleeding identified during the relaparotomy * *N* = 89 cases, in two cases there was more than one source

Figure 2 presents the source of bleeding found during the relaparotomy. The most common source was the uterine scar (31%), with the source of bleeding not found in 30% of the cases.

## Discussion

The present comparative study evaluated the incidence, risk factors, and indications for relaparotomy following a CD during the puerperium in a single-tertiary, university-affiliated, medical center. Our key findings were: (1) The incidence of relaparotomy was 0.46%. (2) Risk factors for relaparotomy included uterine anomalies, uterine fibroids, ART conception, multiple pregnancy, HDP, placental abruption, CD during the second stage of labor, increased surgery duration, complicated CD, and excessive bleeding during the CD and use of bleeding control measures. (3) Most of the relaparotomies occurred during the first 24 h after the CD and the most common indication was suspected intra-abdominal bleeding. (4) The most common finding in the relaparotomy was bleeding from the uterine scar, and the most common intervention was control of the source of bleeding.

The incidence of relaparotomy during the study period was approximately 1:200 CDs, which is in accordance with previous studies describing a rate ranging between 0.23 and 0.7% [5, 7, 15, 16].

In our study, women who conceived by ART had an increased risk for relaparotomy. This finding remained significant even after controlling for possible confounders related to ART such as multiple pregnancy and HDP [21]. Only one previous study examined the association between ART and relaparotomy [7], and found an increasing trend that did not reach statistical significance (aOR 1.83, 95% CI 0.94–3.59). A possible explanation for this association is the known risk factor for third-stage of labor complications in patients undergoing ART, including a higher risk for PPH [22, 23] which is a risk factor for relaparotomy [15].

A fibroid uterus and Mullerian anomalies were also found to be associated with relaparotomy after a CD. These findings were not examined in previous studies. This association can be explained by the higher risk for PPH seen in a fibroid uterus [24], and the increased risk for placental abruption and adherent placenta in Mullerian anomalies [25].

Furthermore, we identified an association between multiple pregnancies and an increased likelihood of requiring relaparotomy after a CD. Twin gestations frequently contribute to over-distention of the uterus, a recognized factor that heightens the risk of uterine atony and PPH [16]. This observation aligns with our discovery that the second most common indication for relaparotomy was uterine atony.

Placental abruption and HDP were also independent risk factors for relaparotomy after a CD, with both entities previously described as risk factors for a relaparotomy [7, 9, 15]. One postulated explanation for this association is the higher risk for PPH in patients suffering from HDP [26] and from placental abruption [27]. Another possible explanation is the increased risk for coagulation disorders that may result in disseminated intravascular coagulation seen in these patients [28, 29], which was detected in almost 30% of women in the study group.

Additionally, CD during the second stage of labor, and longer duration of the CD were found to be associated with the need for relaparotomy, similar to the findings in previous studies [5, 7, 14, 16]. A previous study found that CDs in the second stage compared to those performed in the first stage are related to increased maternal complications including uterine atony and endometritis [30], which were found as causes for relaparotomy in 22.3% of the cases in our cohort. Longer duration of the CD was previously found to increase the risk of postoperative blood transfusion and infection [31]. Although it was not found to be associated with increased risk for relaparotomy, this could be explained by the relatively small number of patients in their cohort as compared to ours (6565 versus 28,280 women) and that they included cases up to one week after CD.

Additional risk factors for relaparotomy related to CD characteristics were complicated CD and excessive bleeding or use of bleeding control measures. While some of the parameters we included were not previously described in the literature, including the use of TXA during the CD, a hemostatic agent, and a surgical drain, others were previously described as being associated with relaparotomy, including calling a 3rd person for assistance and excessive bleeding in the CD [5]. Furthermore, we found that uterine incision extension was associated with an increased risk of relaparotomy. The uterine incision extension can cause GU injury, which was the indication for relaparotomy in eight patients (6.2%). Our findings emphasize the importance of meticulous surgical techniques to try and minimize the relaparotomy rate and maternal morbidity due to the relaparotomy.

Interestingly, the seniority of the surgeon in the CD was not found to be associated with the risk of undergoing a relaparotomy. This finding aligns with a previous study [16] and is contradictory to some studies [5, 14], which, surprisingly, found higher relaparotomy rates when the primary surgeon was an attending physician. However, their findings might be influenced by selection bias, as surgeries considered more surgically complex are likely assigned to experienced surgeons a priori.

In our cohort, 59.2% of relaparotomy cases occurred during the first 24 h after the CD. Previous studies found similar results with 51.2–61.7% of relaparotomy cases occurring in the first 24 h [5, 15, 32], in contrast to the 19% found by Huras et al. [17]. However, the latter study did not specify the indication for the relaparotomy according to the timing from the CD, and hence it is difficult to explore this difference in rates. The main indication for relaparotomy was bleeding in 68.5% of the cases (including intra-abdominal bleeding, subcutaneous hematoma, etc.). These results are compatible with previous studies that found bleeding to be the greatest risk factor for relaparotomy [15, 16].

Regarding the source of bleeding, in 30% of the relaparotomies that occurred for suspected bleeding, the source was not found. While one previous study reported that in 60.7% of the cases, no source was found [14], others reported results similar to ours, with no identifiable source in 12.5-33% of cases [7, 19, 20]. We postulate that the high variation between these studies could be explained by the relatively low number of cases in those studies, ranging between 28 and 64.

Regarding maternal outcomes, there were no cases of maternal death, similar to previous studies [5, 7, 16]. A second relaparotomy was required in 9 women (6.9%), similar to the 6.2% previously described [7], and lower than the 19.7% described by Seal etl al [33]. . In total, 13 women (10%) underwent a hysterectomy (six patients in the first relaparotomy and seven patients in the second relaparotomy). This finding is similar to previous studies which described rates between 7 and 10.6% [16, 19, 20, 33] and is lower than the 33.3% found by Kessous et al. [15]. This discrepancy could be explained by the timing of their study which took place between 1989 and 2009, possibly representing a different approach towards hysterectomy. Another possible explanation could be the rate of primiparous women in the study group, which was 10% in the study by Kessous et al. [15], and 36.2% in our study.

The rate of future deliveries in the relaparotomy group was significantly lower compared to the control group. The rate of future deliveries after relaparotomy has not been described in previous studies, whilst a previous study found that women who suffered from complications after uterine rupture had lower rates of future deliveries as compared to women who did not suffer from complications [34]. Even after excluding the cases of hysterectomy, there was a higher rate of future deliveries at our institution in the control group. There are some possible explanations for these findings. First, women who suffered from complications in their previous delivery may opt to deliver at a different hospital in their following delivery. Another possible explanation could be a higher rate of post-traumatic stress disorder following childbirth (PTSD-FC) in the relaparotomy group, causing these women to subsequently avoid future pregnancies. A previous study found higher rates of PTSD-FC in deliveries complicated by distressing events [35]. Subsequent investigations should aim to elucidate whether this discrepancy is attributed to psychological factors or to biological influences, such as infertility resulting from pelvic adhesions.

Our study has several strengths. Firstly, it is the largest series regarding relaparotomy following a CD reported to date, enabling to explore more precisely possible risk factors for relaparotomy and maternal outcomes. Secondly, we examined a variety of antepartum and intrapartum factors not previously described in the literature, including Mullerian anomalies and TXA use during the CD. Lastly, in our study, we included women during the entire postpartum period, and hence were able to provide more accurate details.

Our study is not without limitations. The study was conducted at a single tertiary medical center in Israel, and the study cohort exhibited a predominantly homogeneous profile. Consequently, the generalizability of our findings to other populations may be limited. Furthermore, due to the retrospective nature of our study, some parameters could not be assessed, such as the time interval from the last LMWH and aspirin dose and the CD. Additionally, we recorded cases that had a CD and a relaparotomy at our institution, but could not assess if there were cases that had the CD at our hospital and subsequently underwent additional surgical intervention at a different hospital.

In conclusion, relaparotomy following a CD is a rare event that confers significant maternal complications and has numerous identifiable risk factors. Our findings could potentially aid clinicians in proactively identifying women at risk of requiring a relaparotomy after a CD, thereby contributing to the mitigation of associated morbidity.

### Electronic supplementary material

Below is the link to the electronic supplementary material.

Supplementary Material 1

## Data Availability

The datasets used and/or analyzed during the current study are available from the corresponding author upon reasonable request.

## Abbreviations

aOR: Adjusted odds ratio
ART: Assisted reproductive technology
CD: Cesarean delivery
EBL: Estimated blood loss
GDM: Gestational diabetes mellitus
HDP: Hypertensive disorders of pregnancy
ICU: Intensive care unit
LMWH: Low molecular weight heparin
OR: Operation room
PAS: Placenta accreta spectrum
PPH: Postpartum hemorrhage
PTSD-FC: Post-traumatic stress disorder following childbirth
TXA: Tranexamic acid
