# Supplementary material for: Risk factors for relaparotomy after a cesarean delivery: a case-control study
Source: BMC Pregnancy Childbirth. 2024 Apr 17;24:284. doi: 10.1186/s12884-024-06455-6 (PMC11022349; doi:10.1186/s12884-024-06455-6)
Supplement: Supplementary file 1 — Supplementary Material 1 [file 12884_2024_6455_MOESM1_ESM.docx]

**Table 1** Multivariable logistic regression of factors associated with a relaparotomy following a cesarean delivery

| Variable | aOR (95% CI) | P-value |
| --- | --- | --- |
| Mullerian anomalies | 3.33 (1.08-10.24) | **0.036** |
| Uterine fibroids | 3.17 (1.11-9.05) | **0.031** |
| ART conception | 4.8 (2.28-10.1) | **<0.001** |
| Multiple pregnancy | 4.1 (1.43-11.79) | **0.009** |
| HDP | 3.46 (1.29-9.3) | **0.014** |
| LMWH use during pregnancy | 2.75 (0.91-8.28) | 0.073 |
| Pre-term delivery | 0.98 (0.42-2.31) | 0.968 |
| Placental abruption | 4.62 (1.09-19.59) | **0.038** |
| CD during the second stage of labor | 2.54 (1.1-5.88) | **0.029** |
| Non-clear amniotic fluid | 1.11 (0.56-2.2) | 0.772 |
| Birthweight (grams) | 1.03 (0.95-1.23) | 0.563 |
| CD duration | 1.12 (1.1-1.3) | **0.048** |
| Complicated CD^*^ | 1.62 (1.09-3.21) | **0.045** |
| Excessive bleeding or use of bleeding control measures^**^ | 2.23 (1.29-4.12) | **0.012** |

Abbreviations: aOR – Adjusted Odds ratio; CI – confidence interval; ART – Assisted reproductive technology; HDP - Hypertensive disorders of pregnancy; LMWH – Low molecular weight heparin; CD – Cesarean delivery.

* Including – intra-abdominal adhesions, uterine incision extensions, calling for assistance, and bladder injury.

** Including – EBL>1000, TXA use during CD, use of a hemostatic agent, and use of a surgical suction drain
